# Distinct gene expression characteristics in epithelial cell-*Porphyromonas gingivalis* interactions by integrating transcriptome analyses

**DOI:** 10.7150/ijms.33728

**Published:** 2019-09-07

**Authors:** Dongmei Zhang, Jingya Hou, Yun Wu, Yanqing Liu, Rong Li, Tong Xu, Junchao Liu, Yaping Pan

**Affiliations:** 1 Department of Periodontics and Oral Biology, School of Stomatology, China Medical University, Shenyang 110002, China; 2 Department of Periodontics, School of Stomatology, China Medical University, Shenyang 110002, China

**Keywords:** Differentially expressed gene, Epithelial cell, Meta-analysis, Microarray, Periodontitis, *Porphyromonas gingivalis*

## Abstract

*Porphyromonas gingivalis* is a pivotal periodontal pathogen, and the epithelial cells serve as the first physical barrier to defend the host from bacterial attack. Within this host-bacteria interaction, *P. gingivalis* can modify the host immune reaction and adjust the gene expression, which is associated with periodontitis pathogenesis and developing strategies. Herein, a meta-analysis was made to get the differential gene expression profiles in epithelial cells with or without *P. gingivalis* infection. The network-based meta-analysis program for gene expression profiling was used. Both the gene ontology analysis and the pathway enrichment analysis of the differentially expressed genes were conducted. Our results determined that 290 genes were consistently up-regulated in *P. gingivalis* infected epithelial cells. 229 gene ontology biological process terms of up-regulated genes were discovered, including “negative regulation of apoptotic process” and “positive regulation of cell proliferation/migration/angiogenesis”. In addition to the well-known inflammatory signaling pathways, the pathway associated with a transcriptional misregulation in cancer has also been increased. Our findings indicated that *P. gingivalis* benefited from the survival of epithelial cells, and got its success as a colonizer in oral epithelium. The results also suggested that infection of *P. gingivalis* might contribute to oral cancer through chronic inflammation. Negative regulation of the apoptotic process and transcriptional misregulation in cancer pathway are important contributors to the cellular physiology changes during infection development, which have particular relevance to the pathogenesis and progressions of periodontitis, even to the occurrence of oral cancer.

## Introduction

Periodontitis is a ubiquitous inflammatory disease and the primary cause of tooth extraction in adults resulted from infection of periodontopathic bacteria. The host manifests an inflammatory immune response to this bacterial infection. *Porphyromonas gingivalis* is an opportunistic pathogen and mainly colonized in periodontal tissues 1. More and more evidence shows that it is also a pathogen of systemic diseases. *P. gingivalis* may modify gene expression, even host immune response by degrading host cell surface proteins or receptors 2, 3. Within this host-bacteria balance, the gene expression is directly correlated with individual susceptibility and influences the pathogenesis of periodontitis and disease progression. Several studies have suggested that plenty of genes were regulated by a host-protective response in gingival tissue of periodontitis. These related genes are critical components of immunological response, biological behavior, and metabolic signal pathways. Recently Kebschull M *et al.* have employed microarray analyses to speculate the gene expression signatures in periodontitis 4-8. It is proved that microarray could supply more insight into the etiology of periodontitis. The growing microarray data offer some valuable clue for analyzing the host immune response of periodontitis. However, the discrimination against critical genes and relevant pathways based on these reports were limited because of the sample size, differences in research design and reporting methods in the separate studies.

A meta-analysis deals with the transcriptome data by combining the individual microarray study, and counters the above-mentioned disadvantage 9. In the current study, a meta-analysis consisting of three separate microarray datasets was made to distinguish the gene expression profile in the epithelial cells with or without *P. gingivalis* infection*.* The present study gives us a synthesized assessment of the gene expression signatures in the epithelial cells with *P. gingivalis* infection. The differentially expressed genes (DEGs), the gene ontology (GO) terms and the Kyoto Encyclopedia of Genes and Genomes (KEGG) pathways concerned in the transcription signatures were identified 10-13. Our analysis would offer new understanding in the comprehension of periodontitis pathobiology, more information on the design of future research, and more tactics to block the progression of periodontitis.

## Materials and Methods

### Gene expression array data collection

An electronic search was performed in Gene Expression Omnibus (GEO, NCBI) database. Microarray data of the gene expression with the keywords “*Porphyromonas gingivalis*” or “gingival epithelial cells” or “oral epithelial cells” were downloaded. Epithelial cells infected with *P.gingivalis* were considered as “case group”, while non-infected epithelial cells as “control group”. Sample-sourced datasets from epithelial cells infected with other bacteria were excluded. Three separate microarray datasets were enrolled with raw data. The details of these datasets were listed in Table 1.

The basic data were obtained from the 3 individual studies, including GEO accession; sample source; platform; numbers of cases or controls in the array data. Two of the datasets were performed in Affymetrix Human Genome U133 Array. The third dataset was conducted in Affymetrix Human OElncRNAs520855F Array. This analysis contains totally 22 GEO transcriptome datasets for epithelial cells infected with *P.gingivalis* or non-infected control. Microarray data for epithelial cells with *P.gingivalis* infection (n = 11) and non-infected control (n = 11) from GEO transcriptome database (http://www.ncbi. nlm.nih.gov/geo/) were compared for their transcriptome profiles.

The research was authorized by the Ethics Committee of School of Stomatology, China Medical University (Shenyang). They determined the current analysis of the public data set did not require the consents of the patients.

### Differential gene expression analysis

DEGs were identified in epithelial cells with *P.gingivalis* infection or not, the datasets extracted from the individual microarray assays were submitted to the network-based meta-analysis program for gene expression profiling (http://www.inmex.ca/INMEX/), starting with multiple gene expression tables for meta-analysis 13-16. First, the gene ID or probe ID was converted to its identical Entrez ID using the gene/probe conversion tool in INMEX. Second, the data were enrolled, processed, annotated and the intensity measurement of every ID was log_2_ transformed and normalized to zero mean and unit variance. While performing differential expression analysis on individual data set, the False Discovery Rate (FDR) of Benjamini-Hochberg's was set 0.05 in order to adjust the cut-off *P*-value. Third, data integrity was assessed as described previously 17-18. Here we used the batch effect correction option so as to minimize the potential batch effect 19. Then the meta-analysis for DGEs was made exploiting the INMEX web-based computational tool. Statistical analysis was performed by the INMEX program. The combined *P* values were detected according to Fisher's method. A *P* value of less than 0.05 was considered as the criterion of statistically significant in our study. The genes selected were classified based on the grade products of the combined *P*-value. The statistics were downloaded and visual exploration was performed. A Venn diagram was made to compare the difference between the findings of the meta-analysis and the individual study (http://bioinformatics.psb.ugent.be/webtools/Venn/).

### Gene Ontology Biological Process terms and KEGG pathway analysis

Subsequently, we exploited a web-based tool (named Database for Annotation, Visualization, and Integrated Discovery) to conduct the GO Biological Process (GO_BP) terms and KEGG pathway analysis 9, 18, 20. DAVID Version 6.8 was used here. In this database, genes were divided into different classes according to their biological processes or molecular functions. Using this GO analysis we compared the DEGs and distributed them into a functional systematization. Gene Entrez ID was uploaded and analyzed for its GO Biological Process Annotation with functional annotation chart after the identifier was selected. Pathway annotations of the DEGs were acquired from the KEGG database 12, 19. Pathway categories with a Benjamini-corrected *P-*value < 0.05 were utilized to determine a critical analysis. Our figures listed the typical GO biological process terms, which were chosen from the functional annotation charts that were significantly enriched at the top. Representative KEGG pathways chosen from the most significantly enriched charts were shown in our figures.

## Results

### DEGs in infected and non-infected epithelial cells

**Figure 1:**
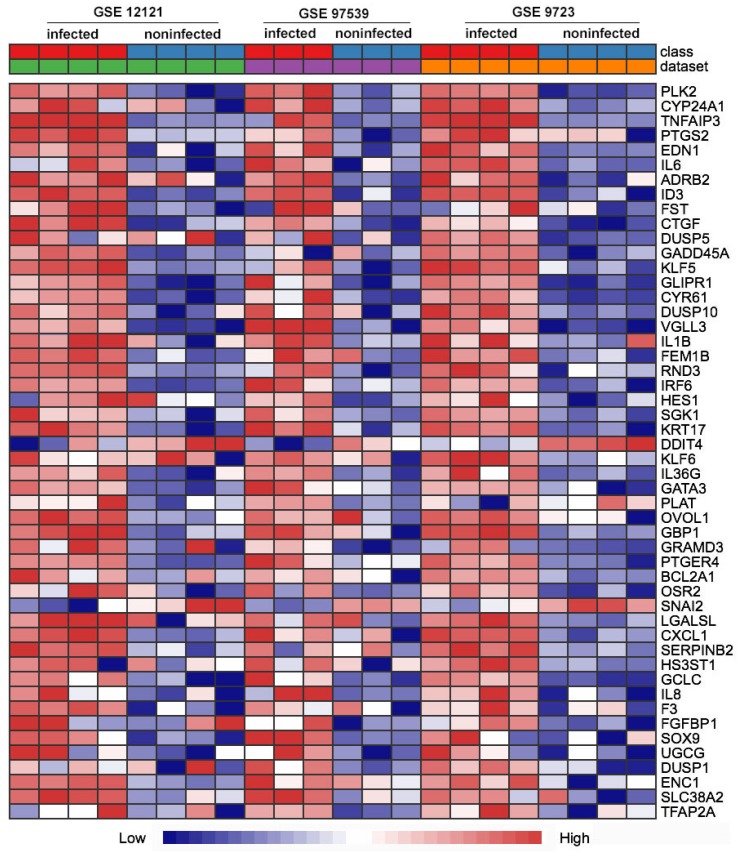
Heat map comparison of the differential gene expression in epithelial cells across the individual datasets with *P. gingivali*s infection or not. Each row means a differentially expressed gene (DEG), each column represents a different GSE data sets. The top 50 misregulated DEGs were included in the map.

First of all, we compared the genome-wide gene expressions of infected and non-infected epithelial cells across microarray datasets. There were 362 genes differentially expressed in the two groups of epithelial cells. Among the 362 DEGs, 290 genes were significantly up-regulated (Table S1), and 72 genes were significantly down-regulated (Table S2). The up-regulated DEGs consisted of *PLK2*, *CYP24A1*, *TNFAIP3*,* PTGS2*,* EDN1*,* IL6*,* GADD45A*, *IL1B*, etc. The top 50 up-regulated DEGs were shown in the heatmap (Fig. 1).

The most consistently up- and down-regulated DEGs of top 20 in epithelial cells, in the comparison of infected and non-infected epithelial cells, were shown in Table 2.

**Figure 2:**
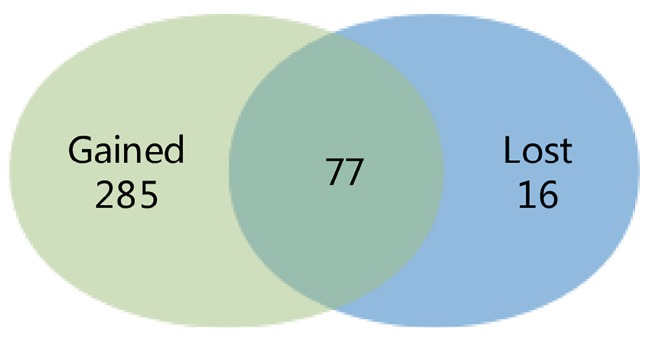
Venn diagram showing the comparison of the results of the meta-analysis and the individual study. The overlying part represents differentially expressed genes (DEGs) determined in epithelial cells in the two investigations, gained DEGs indicate those charactered in the meta-analysis only and lost DEGs indicate those in the individual analysis only.

We compared the difference between the findings of the meta-analysis and the separate study, and a Venn diagram was made. 285 DEGs were distinguished in the meta-analysis only, which called gained DEGs. While only 16 DEGs were found in the separate study only, which called lost DEGs (Fig. 2).

### Functional classification and pathway assignment of DEG

**Figure 3:**
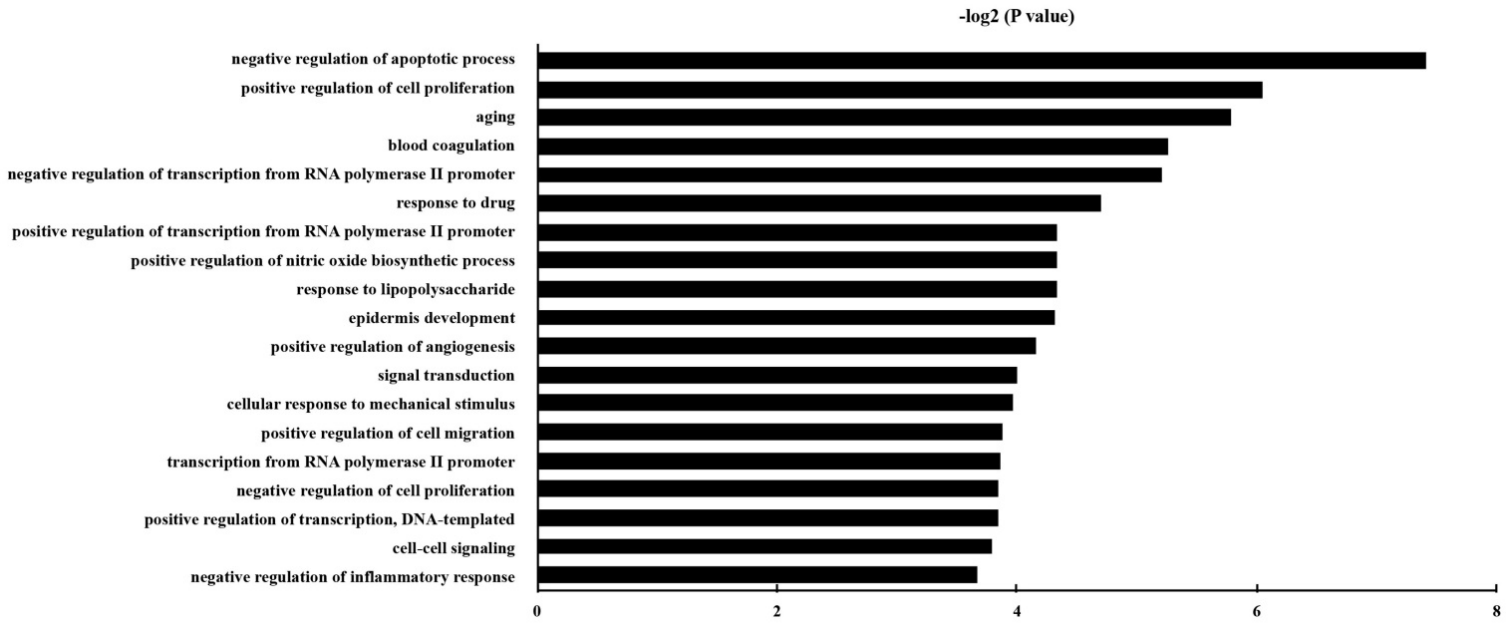
The gene ontology classification of the up-regulated genes in epithelial cells with *P. gingivalis* infection than those without infection. *P* and FDR < 0.05 were considered as the criterion of statistically significant in GO category investigation.

GO analysis was carried out, and the DEGs were classified into different hierarchical categories based on the GO database. 229 GO Biological Process terms of up-regulated genes were discovered (Table S3). It has been shown that the significantly enriched GO terms for the DEGs included “negative regulation of apoptotic process”, “aging”, “positive regulation of cell proliferation”, “positive regulation of cell migration”, and “positive regulation of angiogenesis”. The top 20 enriched GO terms for up-regulated DEG in epithelial cells were shown in Fig. 3.

Here, we paid more attention to the first GO terms, namely negative regulation of apoptotic process. And the involved DEGs associated with up-regulated biological process categories of negative regulation of apoptotic process were listed in Table 3.

**Figure 4:**
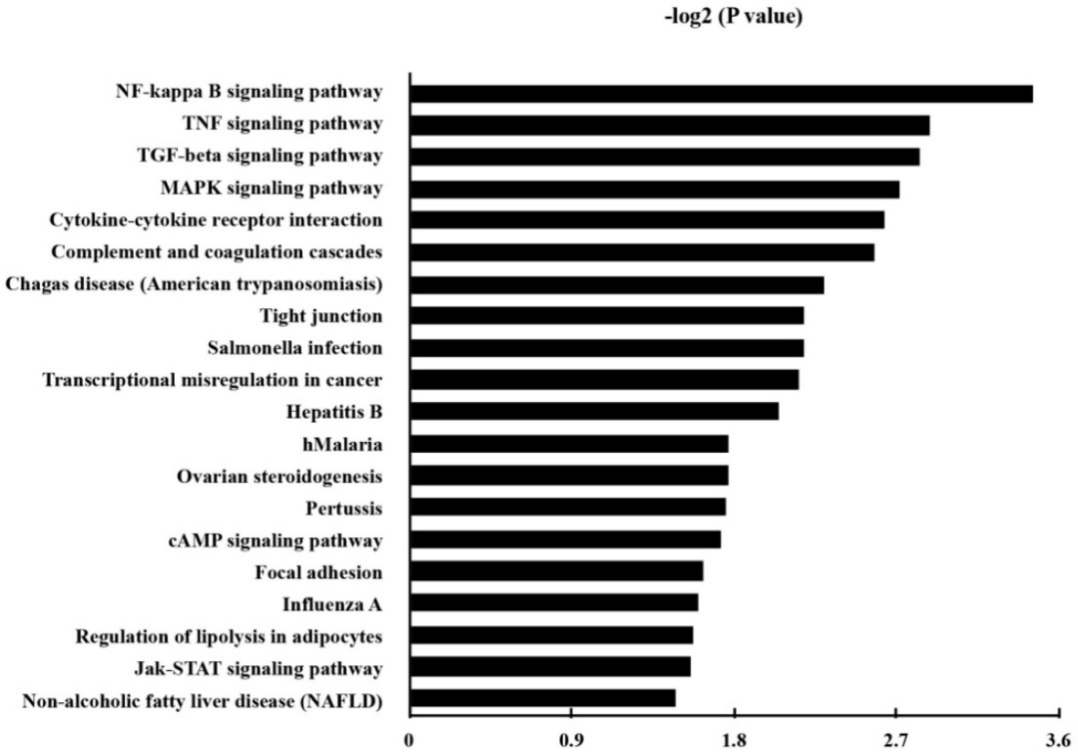
The Kyoto Encyclopedia of Genes and Genomes (KEGG) pathways of the up-regulated genes in *P. gingivalis*-infected cells compared to the non-infected cells. *P* and FDR < 0.05 were considered as the criterion of statistically significant in the pathway analysis.

KEGG pathway analysis was made in order to annotate the functional categories of DEGs as well. Here, 38 up-regulated pathways were found (Table S4). The 20 most significantly up-regulated pathways for DEGs were demonstrated in Fig. 4.

Besides the well-known inflammatory signaling pathways, such as nuclear factor kappa-light-chain-enhancer of activated B cells (NF-κB) signaling pathway, tumor necrosis factor (TNF) signaling pathway, mitogen-activated protein kinase (MAPK) signaling pathway, we found that the pathway associated with a transcriptional misregulation in cancer, Janus kinases/signal transducer and activator of transcription (JAK/Stat) pathway, and transforming growth factor-β (TGF-β) signaling pathway, had also been increased. Representatively, the involved DEGs of the KEGG pathways of transcriptional misregulation in cancer were listed in Table 4.

## Discussion

As a Gram-negative anaerobic bacterium, *P. gingivalis* is one of the most prominent pathogens in the etiology of chronic periodontitis. Epithelial cells are now considered to be the first immune barrier in the pathogenesis of periodontal disease confronted by the microorganism that colonizes or even intrudes mucosal surfaces. The intercommunication of *P. gingivalis* with epithelial cells involves several intricate signaling pathways 21-23. Recognition of the pertinent genes and signal pathways concerned is pivotal, so we could comprehend the cellular and molecular changes during the periodontitis onset and disease progression. Considering *P. gingivalis* can not only invade the local periodontal tissue but also persist in other systems of the human being (for example esophagus tissue, respiratory and vascular), we should be more cautious in the active performance of *P. gingivalis* with epithelial cells.

Now we have obtained the transcriptomic profile with our meta-analysis using several public microarray data. Taking advantage of this enhanced statistical power, our results were more reliable 24-25. We identified the DEGs, the corresponding Biological Process and pathways in *P. gingivalis*-infected epithelial cells based on comparisons to the GO 8,9 and KEGG 12-13 databases, respectively. The present results indicated the changes in gene expression feature modulated by some signal pathways were related to host-bacterial balance.

In our meta-analysis we showed several genes about negative regulation of the apoptotic process, including *BCL2A1*, *BCL2*, *BARD1*, *CFLAR*, were significantly up-regulated in epithelial cells infected with *P. gingivalis* compared to non-infected ones. It is conceived that *P. gingivalis* improves the survival of host cells by directly inhibiting different pro-apoptotic pathways 3, 26-28. Inducing the durability of epithelial cells is crucial for the survival of *P. gingivalis* in these cells. It is reported that this resistance to apoptosis may be related to the BCL2 protein family, including BCL-2 and BAX 29. This suppressed apoptosis during *P. gingivalis* infection was phosphatidylinositol 3 kinase/Protein Kinase B (PI3K/Akt) -dependent, furthermore, the PI3K signal pathway could regulate transcription or post-translational modification of certain BCL2 family member 3.

Our results verified that *P. gingivalis* infection has numerous anti-apoptotic effects on epithelial cells (such as activation of JAK/Stat pathways, NF-κB signal pathway, etc). The diversity of these multiple genes and pathways demonstrates that *P. gingivalis* may affect apoptotic pathways at different levels. Some of the up-regulated genes and pathways surround the mitochondria, which is a critical component of the intrinsic apoptosis process. The analysis of our study, along with others reports, showed that *P. gingivalis* suppressed the apoptosis of epithelial cell partially through the JAK/Stat signal pathway, which regulates the inherent mitochondria cell death mediated by the caspase-dependent apoptotic pathway.

At the meantime, *P. gingivalis* participates in the regulation of the extrinsic apoptotic pathway. Our KEGG pathway analysis demonstrated NF-κB signal pathway was up-regulated. It is reported that NF-κB, together with TNF and FasL, takes its role in the composition of the extrinsic apoptotic process 30. NF-κB activation serves as a primary mechanism to protect cells against apoptotic stimulus such as TNF 31. Current findings and early reports verify that the ability of *P. gingivalis* to benefit the survival of epithelial cells may assist its successful colonization in the oral mucosal epithelium.

Besides, the current analysis found that the transcriptional misregulation in cancer, another KEGG pathway, was up-regulated. Related genes expression, including C-X-C motif chemokine ligand 8 (*CXCL8*), CCAAT/enhancer binding protein epsilon (*CEBPB*), Meis homeobox 1 (*MEIS1*), interleukin 6 (*IL-6*), myeloperoxidase (*MPO*) and plasminogen activator urokinase (*PLAU*) were increased significantly.

*IL6* is regarded as a tumor-promoting cytokine in diverse kinds of cancer including oral squamous cell carcinoma (OSCC) 32-35. It is considered as one of the most promising markers for early diagnosis of tongue squamous cell carcinoma, which was the most usual kind of OSCC. Geng *et al.* revealed that both *CEBPB and IL6* were up-regulated in late OSCC and *CEBPB* was activated as an upstream regulator of *IL6*. Moreover, *CEBPB* was recently identified as one of the master regulators in cancer biology 35,36.

Nowadays, more researches have reported a significant increase of *CXCL8* and its mRNA in *P. gingivalis*-infected epithelial cells or oral squamous cells 37-39. Ha's study found that infection by *P. gingivalis* stimulated the tumorigenic features and invasiveness of oral squamous cells, which was correlated with enhanced expression of *CXCL8* 37. PLAU, the serine protease, has been indicated in promoting pericellular proteolysis and oncogenic signal transduction in cancer cells. Yoshizawa implied that cases with PLAU/PLAU receptor/maspin expression should be considered as the high risk of poor prognosis of OSCC, which may lead to severe invasiveness, as well as the increased risk for cervical lymph node metastasis 40.

In addition, over-expression of MEIS1 in acute leukemia has been observed 41, which is related to the shorted latency and accelerated progression of acute leukemia 42. The enhancer E9 mediates an auto-regulatory loop which contributes to the consistent expression of MEIS1 in leukemia. It was reported that MPO levels were dramatically enhanced in the bronchoalveolar lavage fluid and positively related to the carcinogenicity of inflammatory in lung cancer formation 43-44. MPO is conceived as a critical mediator of inflammatory-promoted tumorigenesis, and its pro-tumor effects may take place in the window just after tumor onset in the lung 45.

So far, several studies have attempted to elucidate the role of *P. gingivalis* in contribution to oral cancer. Previous research by our group showed *P. gingivalis* enhanced the cell proliferation, migration, and invasive ability of human oral epithelial cells. Thus it promoted the cells tumorigenic properties 2. Gonda *et al.* reported that chronic inflammation induced by bacterial infection drove carcinogenesis, involving induction, progression, invasion, and metastasis 33. It is believed that the inflammatory microenvironment was an integral part of oral cancer 2,32-35,46,47.

Our KEGG pathway analysis further confirmed *P. gingivalis* played a role in promoting tumor transformation in some inflammatory conditions. Here pathways enrichment investigation showed that several pathways were enhanced significantly, including the NF-κB pathway, TNF pathway, TGF-beta pathway, MAPK signal pathway, and cytokine-cytokine receptor interaction. These inflammation-related signaling pathways were listed as the top 5 of our KEGG pathway analysis results. Here we believe that this chronic bacteria infection should be identified as probable effector which may result in oral cancer.

Generally speaking, the current meta-analysis using microarray datasets offered us an outlook about the gene expression characteristics in epithelial cells infected with *P.gingivalis*. The bioinformatics analyses of the up-regulated genes showed us that the GO terms of the negative regulation of apoptotic process, several inflammation-related signaling pathways, and the transcriptional misregulation in cancer were significantly enriched. It provided us some new sights into the alteration in cytopathogenicity that took place in the process of infection, which had particular relevance to periodontitis pathogenesis and developing strategies. Meanwhile, it provided more evidence to support the view that *P.gingivalis* might be related to the occurrence and development of OSCC.

## Supplementary Material

Supplementary table: All the up-regulated genes were listed in supplementary table S1; All the down-regulated genes were listed in supplementary table S2; GO Biological Process terms of up-regulated genes were listed in supplementary table S3; KEGG pathway of up-regulated genes were listed in supplementary table S4.

## Figures and Tables

**Table 1:** Information on transcriptome datasets used in the present analysis

No.	GEO accession	Sample source	Platform	Sample size Case Control	Reference
1	GSE 97539	human immortalized oral epithelial cell	GPL22516, [OElncRNAs520855F] Affymetrix Human OElncRNAs520855F Array	3 3	2
2	GSE 12121	gingival epithelial HIGK cell	GPL570,[HG-U133_Plus_2] Affymetrix Human Genome U133 Plus 2.0 Array	4 4	7
3	GSE 9723	gingival epithelial HIGK cell	GPL96, [HG-U133A] Affymetrix Human Genome U133A Array	4 4	8

**Table 2:** Top 20 up-regulated and down-regulated differentially expressed genes (DEGs) in *P. gingivalis*-infected epithelial cells compared to non-infected cells

Entrez ID	Gene Symbol	Combined T stat	Combined P value	Entrez ID	Gene Symbol	Combined T stat	Combined P value
*Up-regulated genes*				*Down-regulated genes*			
10769	PLK2	-75.763	<1.0E-10	54541	DDIT4	39.654	<1.0E-04
1591	CYP24A1	-59.072	<1.0E-07	6591	SNAI2	35.697	<0.001
7128	TNFAIP3	-54.414	<1.0E-06	7071	KLF10	28.802	<0.01
5743	PTGS2	-52.335	<1.0E-06	26276	VPS33B	26.858	<0.01
1906	EDN1	-52.685	<1.0E-06	11067	C10orf10	26.815	<0.01
3569	IL6	-53.09	<1.0E-06	6574	SLC20A1	26.779	<0.01
154	ADRB2	-51.163	<1.0E-06	55844	PPP2R2D	26.31	<0.01
3399	ID3	-50.168	<1.0E-06	117247	SLC16A10	26.115	<0.01
10468	FST	-49.696	<1.0E-06	10224	ZNF443	24.8	<0.01
1490	CTGF	-49.837	<1.0E-06	4016	LOXL1	24.512	<0.01
1847	DUSP5	-49.125	<1.0E-06	79759	ZNF668	24.307	<0.01
1647	GADD45A	-48.243	<1.0E-06	10561	IFI44	24.232	<0.01
688	KLF5	-47.733	<1.0E-05	27034	ACAD8	24.179	<0.01
11010	GLIPR1	-47.307	<1.0E-05	54014	BRWD1	24.036	<0.01
3491	CYR61	-46.897	<1.0E-05	9778	KIAA0232	23.467	<0.01
11221	DUSP10	-46.685	<1.0E-05	5154	PDGFA	23.45	<0.01
389136	VGLL3	-46.265	<1.0E-05	8772	FADD	23.374	<0.01
3553	IL1B	-44.656	<1.0E-05	1390	CREM	23.33	<0.01
10116	FEM1B	-44.385	<1.0E-05	211	ALAS1	23.256	<0.01
390	RND3	-43.556	<1.0E-05	283871	PGP	23.211	<0.01

Left columns: up-regulated DEGs, combined T stat < 0; Right columns: down-regulated DEGs, Combined T stat > 0

**Table 3:** Top 10 of differentially expressed genes list associated with up-regulated gene ontology biological process categories of negative regulation of the apoptotic process

Entrez ID	Gene Symbol	Gene Name
597	BCL2A1	BCL2 related protein A1
596	BCL2	BCL2, apoptosis regulator
580	BARD1	BRCA1 associated RING domain 1
8837	CFLAR	CASP8 and FADD like apoptosis regulator
2627	GATA6	GATA binding protein 6
55679	LIMS2	LIM zinc finger domain containing 2
1482	NKX2-5	NK2 homeobox 5
5292	PIM1	Pim-1 proto-oncogene, serine/threonine kinase
6662	SOX9	SRY-box 9
301	ANXA1	annexin A1

**Table 4:** Top 10 of differentially expressed genes list associated with up-regulated Kyoto Encyclopedia of Genes and Genomes pathways of transcriptional misregulation in cancer

Entrez ID	Gene Symbol	Gene Name
597	BCL2A1	BCL2 related protein A1
3576	CXCL8	C-X-C motif chemokine ligand 8
1053	CEBPE	CCAAT/enhancer binding protein epsilon
4211	MEIS1	Meis homeobox 1
3569	IL6	interleukin 6
7403	KDM6A	lysine demethylase 6A
4353	MPO	myeloperoxidase
5327	PLAT	plasminogen activator, tissue type
5328	PLAU	plasminogen activator, urokinase
4609	MYC	v-myc avian myelocytomatosis viral oncogene homolog

## Abbreviations

DEGs: differentially expressed genes
FDR: False Discovery Rate
GEO: Gene Expression Omnibus
GO: gene ontology
INMEX: integrative meta-analysis of expression data
KEGG: Kyoto Encyclopedia of Genes and Genomes
NF-κB: nuclear factor kappa-light-chain-enhancer of activated B cells
OSCC: oral squamous cell carcinoma
TGF-β: transforming growth factor-beta
TNF: tumor necrosis factor
