# Supplementary material for: Distinct gene expression characteristics in epithelial cell-Porphyromonas gingivalis interactions by integrating transcriptome analyses
Source: Int J Med Sci. 2019 Sep 7;16(10):1320–7. doi: 10.7150/ijms.33728 (PMC6818190; doi:10.7150/ijms.33728)
Supplement: Supplementary file 1 — Supplementary table: All the up-regulated genes were listed in supplementary table S1; All the down-regulated genes were listed in supplementary table S2; GO Biological Process terms of up-regulated genes were listed in supplementary table S3; KEGG pathway of up-regulated genes were listed in supplementary table S4. [file ijmsv16p1320s1.pdf]

Table S1 meta_sig_genes_metap UG 290 DEG

EntrezID	Name	Combine	CombinedPval
10769	PLK2	-75.763	0000E-11
1591	CYP24A1	-59.072	0000E-08
7128	TNFAIP3	-54.414	0000E-07
3569	IL6	-53.09	0000E-07
1906	EDN1	-52.685	0000E-07
5743	PTGS2	-52.335	0000E-07
154	ADRB2	-51.163	0000E-07
3399	ID3	-50.168	0000E-07
1490	CTGF	-49.837	0000E-07
10468	FST	-49.696	0000E-07
1847	DUSP5	-49.125	0000E-07
1647	GADD45	-48.243	0000E-07
688	KLF5	-47.733	0000E-06
11010	GLIPR1	-47.307	0000E-06
3491	CYR61	-46.897	0000E-06
11221	DUSP10	-46.685	0000E-06
389136	VGLL3	-46.265	0000E-06
3553	IL1B	-44.656	0000E-06
10116	FEM1B	-44.385	0000E-06
390	RND3	-43.556	0000E-06
3664	IRF6	-43.364	0000E-06
3280	HES1	-41.743	0000E-05
6446	SGK1	-40.336	0000E-05
3872	KRT17	-40.316	0000E-05
1316	KLF6	-39.373	0000E-05
56300	IL36G	-39.371	0000E-05
2625	GATA3	-38.924	0000E-05
5327	PLAT	-38.318	0000E-05
5017	OVOL1	-37.741	0000E-05
2633	GBP1	-36.633	0000E-05
65983	GRAMD3	-36.627	0000E-05
116039	OSR2	-36.28	0000E-05
5734	PTGER4	-36.242	0000E-05
597	BCL2A1	-36.23	0000E-05
29094	LGALSL	-35.556	0012256
5055	SERPINB	-35.305	0013192
2919	CXCL1	-35.29	0013192
9957	HS3ST1	-35.205	0013389
2729	GCLC	-35.145	0013417
3576	IL8	-35.097	0013417
2152	F3	-34.954	0014021
9982	FGFBP1	-34.651	0015813
8507	ENC1	-33.998	0020666
1843	DUSP1	-33.959	0020666
6662	SOX9	-33.93	0020666
7357	UGCG	-33.879	0020666
54407	SLC38A2	-33.857	0020666
7020	TFAP2A	-33.778	0021021
6659	SOX4	-33.298	0025847
27071	DAPP1	-33.014	0028984
8660	IRS2	-32.945	0029382
9076	CLDN1	-32.679	0003269
5328	PLAU	-32.608	0033175
79669	C3orf52	-32.484	0034544
4929	NR4A2	-32.23	0038251
7538	ZFP36	-31.979	0.000423
5450	POU2AF1	-31.773	0045822
23766	GABARAP	-31.605	0048759
22914	KLRK1	-31.062	0061923
51421	AMOTL2	-30.902	0065675
1825	DSC3	-30.713	0070623
7159	TP53BP2	-30.659	0071309
9152	SLC6A5	-30.485	0076173
23529	CLCF1	-30.404	0077933
6722	SRF	-30.366	0078135
8843	HCAR3	-30.271	0.000805
3854	KRT6B	-29.903	0094285
7764	ZNF217	-29.774	0098715
4616	GADD45	-29.66	0010269
694	BTG1	-29.403	0011423
4430	MYO1B	-29.052	0013272
9021	SOCS3	-28.989	0013487
0506658	OCLN	-28.864	0014105
25956	SEC31B	-28.333	0017614
51085	MLXIPL	-28.21	0018416
2150	F2RL1	-27.97	0020049
10979	FERMT2	-27.945	0020049
221823	PRPS1L1	-27.934	0020049
1948	EFNB2	-27.921	0020049
1942	EFNA1	-27.715	0.00218
3861	KRT14	-27.667	0022031
7168	TPM1	-27.604	0022417
5329	PLAUR	-27.513	0023119
7403	KDM6A	-27.485	0023156
23353	SUN1	-27.458	0023176
1022	CDK7	-27.426	0023258
5292	PIM1	-27.147	0026196
80352	RNF39	-27.053	0027066
7056	THBD	-26.906	0028677
4081	MAB21L1	-26.487	0033395
3868	KRT16	-26.367	0034624
696	BTN1A1	-26.365	0034624
57817	HAMP	-26.256	0035687
79098	C1orf116	-26.162	0036909
29930	PCDHB1	-26.019	0.003867
0169750	PRINS	-25.685	0.004474
2250	FGF5	-25.616	0045348
148022	TICAM1	-25.614	0045348
1472	CST4	-25.481	0047778
9590	AKAP12	-25.386	0049478
3777	KCNK3	-25.294	0051168
1075	CTSC	-25.113	0054652
4609	MYC	-25.112	0054652
313	AOAH	-25.053	0055677
55679	LIMS2	-24.803	0.00615
1793	DOCK1	-24.767	0.006189
10627	MYL12A	-24.606	0066106
760	CA2	-24.548	0066869
9052	GPRC5A	-24.537	0066869
3037	HAS2	-24.508	0066869
9314	KLF4	-24.412	0069301
639	PRDM1	-24.36	0070418
2627	GATA6	-24.246	0072339
10447	FAM3C	-24.237	0072339
54101	RIPK4	-24.143	0073756
23078	KIAA056	-24.137	0073756
2294	FOXF1	-24.122	0073756
10398	MYL9	-24.074	0074832
10741	RBBP9	-24.051	0075029
2155	F7	-24.007	0075458
3909	LAMA3	-23.835	0081103
1543	CYP1A1	-23.567	0090804
4211	MEIS1	-23.558	0090804
7286	TUFT1	-23.374	0096044
55859	BEX1	-23.335	0096626
5099	PCDH7	-23.176	0.010106
29881	NPC1L1	-23.173	0.010106
23237	ARC	-23.012	0.010807
9388	LIPG	-22.979	0.010897
3400	ID4	-22.906	0.01115
4335	MNT	-22.9	0.01115
2550	GABBR1	-22.882	0.011172
301	ANXA1	-22.733	0.011853
53635	PTOV1	-22.681	0.012014
57053	CHRNA1	-22.635	0.012195
7050	TGIF1	-22.589	0.012332
580	BARD1	-22.583	0.012332
9048	ARTN	-22.544	0.012444
7465	WEE1	-22.536	0.012444
54626	HES2	-22.52	0.012453
238	ALK	-22.502	0.012479
64063	PRSS22	-22.473	0.012494
57132	CHMP1B	-22.434	0.012524
10261	IGSF6	-22.427	0.012524
1809	DPYSL3	-22.426	0.012524
4633	MYL2	-22.415	0.012524
10626	TRIM16	-22.369	0.012719
80342	TRAF3IP	-22.356	0.012719
115207	KCTD12	-22.325	0.012825
54438	GFOD1	-22.294	0.012935
2355	FOSL2	-22.266	0.013023
9450	LY86	-22.213	0.013242
5912	RAP2B	-22.205	0.013242
9414	TJP2	-22.18	0.013318
23339	VPS39	-22.107	0.013696
8200	GDF5	-21.993	0.014314
2172	FABP6	-21.965	0.014332
6585	SLIT1	-21.959	0.014332
5676	PSG7	-21.897	0.014623
3552	IL1A	-21.891	0.014623
3459	IFNGR1	-21.871	0.014679
6575	SLC20A2	-21.757	0.015216
800	CALD1	-21.686	0.01562
79170	PRR15L	-21.663	0.01563
3493	IGHA1	-21.646	0.015674
8416	ANXA9	-21.6	0.015692
79586	CHPF	-21.587	0.015692
5076	PAX2	-21.56	0.015728
201134	CEP112	-21.502	0.016067
81931	ZNF93	-21.484	0.01612
30844	EHD4	-21.459	0.016228
9465	AKAP7	-21.408	0.016521
7057	THBS1	-21.381	0.016646
2158	F9	-21.324	0.017006
5366	PMAIP1	-21.29	0.017188
440915	POTEKP	-21.277	0.017206
83937	RASSF4	-21.185	0.017857
752014	CEMP1	-21.12	0.018219
1822	ATN1	-21.059	0.018643
168544	ZNF467	-20.964	0.019285
4884	NPTX1	-20.95	0.019314
79740	ZBBX	-20.94	0.019314
5783	PTPN13	-20.907	0.019522
6698	SPRR1A	-20.721	0.021151
107	ADCY1	-20.696	0.021295
483	ATP1B3	-20.682	0.021334
27134	TJP3	-20.661	0.021433
8609	KLF7	-20.645	0.021455
4214	MAP3K1	-20.614	0.021606
7380	UPK3A	-20.54	0.022246
108	ADCY2	-20.51	0.022416
80271	ITPKC	-20.504	0.022416
8837	CFLAR	-20.446	0.022808
3484	IGFBP1	-20.43	0.022808
27115	PDE7B	-20.43	0.022808
55203	LGI2	-20.425	0.022808
2312	FLG	-20.417	0.022808
11259	FILIP1L	-20.318	0.023508
6590	SLPI	-20.317	0.023508
268	AMH	-20.313	0.023508
357	SHROOM	-20.248	0.024112
6247	RS1	-20.221	0.024309
51286	CEND1	-20.207	0.024344
2248	FGF3	-20.165	0.024616
1545	CYP1B1	-20.136	0.024848
11250	GPR45	-20.117	0.024954
27010	TPK1	-20.065	0.025341
4993	OR2C1	-20.051	0.025403
1465	CSRP1	-20.04	0.025421
84159	ARID5B	-20.031	0.025426
4353	MPO	-20.021	0.025436
27042	DIEXF	-19.895	0.02683
1053	CEBPE	-19.881	0.026891
81628	TSC22D4	-19.822	0.027397
1068	CETN1	-19.81	0.027435
79885	HDAC11	-19.765	0.027899
58483	LINC004	-19.735	0.028143
50835	TAS2R9	-19.728	0.028143
1482	NKX2-5	-19.692	0.028499
1609	DGKQ	-19.641	0.028935
2972	BRF1	-19.633	0.028935
23022	PALLD	-19.594	0.029267
4070	TACSTD2	-19.576	0.029351
84909	C9orf3	-19.523	0.029949
1960	EGR3	-19.505	0.02998
79037	PVRIG	-19.504	0.02998
7091	TLE4	-19.491	0.03004
1439	CSF2RB	-19.435	0.030571
441151	TMEM151	-19.426	0.030571
22898	DENND3	-19.414	0.030571
63967	CLSPN	-19.411	0.030571
55317	AP5S1	-19.403	0.030571
83700	JAM3	-19.321	0.031465
8698	S1PR4	-19.316	0.031465
4745	NELL1	-19.258	0.032052
4071	TM4SF1	-19.214	0.032477
28234	SLCO1B3	-19.198	0.032591
3081	HGD	-19.189	0.03261
2354	FOSB	-19.177	0.03261
29974	A1CF	-19.173	0.03261
26959	HBP1	-19.138	0.033014
26040	SETBP1	-19.12	0.033163
1039	CDR2	-19.113	0.033163
962	CD48	-19.018	0.034391
375449	MAST4	-18.952	0.035176
3725	JUN	-18.912	0.035579
55478	PRO2012	-18.895	0.035727
1832	DSP	-18.876	0.035801
9182	RASSF9	-18.875	0.035801
58473	PLEKHB1	-18.772	0.036896
596	BCL2	-18.65	0.03846
3448	IFNA14	-18.556	0.039491
3249	HPN	-18.548	0.039502
57165	GJC2	-18.541	0.039502
79097	TRIM48	-18.525	0.039658
26509	MYOF	-18.462	0.04041
57213	SPRYD7	-18.432	0.040698
10957	PNRC1	-18.392	0.041315
5268	SERPINB	-18.372	0.041556
79962	DNAJC22	-18.352	0.041715
80223	RAB11FI	-18.35	0.041715
54769	DIRAS2	-18.307	0.041999
79825	CCDC48	-18.304	0.041999
1846	DUSP4	-18.302	0.041999
8412	BCAR3	-18.301	0.041999
6876	TAGLN	-18.292	0.041999
0289255	LOC1002	-18.285	0.041999
7293	TNFRSF4	-18.283	0.041999
23053	KIAA091	-18.241	0.042526
80258	EFHC2	-18.205	0.042793
140	ADORA3	-18.173	0.043277
25976	TIPARP	-18.129	0.044026
51706	CYB5R1	-18.091	0.044375
7032	TFF2	-18.07	0.04456
10046	MAMLD1	-18.069	0.04456
80310	PDGFD	-18.038	0.045045
54891	INO80D	-18.026	0.045142
150160	CCT8L2	-18.021	0.045142
64760	FAM160B	-18.006	0.045303
5024	P2RX3	-17.985	0.045613
59335	PRDM12	-17.968	0.045775
80149	ZC3H12A	-17.964	0.045775
4352	MPL	-17.92	0.046549
60506	NYX	-17.867	0.047406
1768	DNAH6	-17.858	0.04746
57172	CAMK1G	-17.843	0.04747
3791	KDR	-17.836	0.04747
9398	CD101	-17.802	0.047991
10320	IKZF1	-17.793	0.048064
956	ENTPD3	-17.782	0.048157

Table S2 meta_sig_genes_metap DG 72 DEG				
EntrezID	Name	Combine	CombinedPval	
54541	DDIT4	39.654	0000E-05	
6591	SNAI2	35.697	0011782	
7071	KLF10	28.802	0014331	
26276	VPS33B	26.858	0029001	
11067	C10orf10	26.815	0029282	
6574	SLC20A1	26.779	0029459	
55844	PPP2R2D	26.31	0035159	
117247	SLC16A1	26.115	0037347	
10224	ZNF443	24.8	0.00615	
4016	LOXL1	24.512	0066869	
79759	ZNF668	24.307	0071582	
10561	IFI44	24.232	0072339	
27034	ACAD8	24.179	0073529	
54014	BRWD1	24.036	0075029	
9778	KIAA023	23.467	0093984	
5154	PDGFA	23.45	0094071	
8772	FADD	23.374	0096044	
1390	CREM	23.33	0096626	
211	ALAS1	23.256	0099284	
283871	PGP	23.211	.010065	
668	FOXL2	22.725	.011853	
80055	PGAP1	22.475	.012494	
9997	SCO2	21.986	.014314	
4857	NOVA1	21.82	.014944	
5834	PYGB	21.778	.015151	
10628	TXNIP	21.676	0.01562	
80304	C2orf44	21.622	.015692	
27346	TMEM97	21.616	.015692	
200424	TET3	21.596	.015692	
55654	TMEM12	21.574	.015704	
8793	TNFRSF1	21.123	.018219	
8894	EIF2S2	20.985	0.01919	
56937	PMEPA1	20.639	.021455	
643376	BTBD18	20.369	.023212	
3421	IDH3G	20.199	.024344	
23221	RHOBTB	20.101	.025035	
57396	CLK4	19.823	.027397	
79778	MICALL2	19.635	.028935	
1594	CYP27B1	19.591	.029267	
10948	STARD3	19.408	.030571	
55143	CDCA8	19.386	.030706	
9524	TECR	19.265	.032052	
114625	ERMAP	19.251	.032052	
10363	HMG20A	19.017	.034391	
79908	BTNL8	18.981	.034846	
79888	LPCAT1	18.924	.035499	
115353	LRRC42	18.847	.036146	
63917	GALNT11	18.837	.036189	
23250	ATP11A	18.805	.036589	
9015	TAF1A	18.785	0.03679	
9416	DDX23	18.745	0.03722	
79819	WDR78	18.664	0.03846	
54948	MRPL16	18.652	0.03846	
7086	TKT	18.637	.038566	
8464	SUPT3H	18.588	.039281	
11320	MGAT4A	18.582	.039281	
58529	MYOZ1	18.563	.039478	
8225	GTPBP6	18.499	.039991	
10253	SPRY2	18.488	.040069	
53916	RAB4B	18.433	.040698	
30012	TLX3	18.28	.041999	
10885	WDR3	18.239	.042526	
65987	KCTD14	18.232	.042526	
51167	CYB5R4	18.209	.042793	
1581	CYP7A1	18.109	.044191	
10123	ARL4C	18.107	.044191	
10467	ZNHIT1	17.873	.047406	
5900	RALGDS	17.844	0.04747	
648	BMI1	17.833	0.04747	
55039	TRMT12	17.744	.048861	
55695	NSUN5	17.725	0.04914	
79762	C1orf115	17.705	.049451	

Table S3 GO Biological Process terms of up-regulated genes

Category	Term	Count	%	PValue	Genes	List Total	Pop Hits	Pop Total	Fold Enri	Bonferroi	Benjamir	FDR
GOTERM_	GO:0043066~negative regulation of apoptotic process	26	4137931	3.863223	597, 596	259	455	16792	3.704798	7.996552	7.996552	6.668797
GOTERM_	GO:0008284~positive regulation of cell proliferation	24	8.275862	9.142165	3037, 99	259	466	16792	3.339088	0.001890	9.457670	0.001578
GOTERM_	GO:0007568~aging	14	4.827586	1.643999	3725, 59	259	165	16792	5.501064	0.003397	0.001133	0.002837
GOTERM_	GO:0007596~blood coagulation	14	4.827586	5.512988	2158, 26	259	184	16792	4.933019	0.011347	0.002848	0.009516
GOTERM_	GO:0000122~negative regulation of transcription from RNA polymerase II pro	29	10	6.335871	10320, 7	259	720	16792	2.611368	0.013029	0.002619	0.010936
GOTERM_	GO:0042493~response to drug	17	5.862068	2.003452	3725, 59	259	304	16792	3.625584	0.040623	0.006888	0.034578
GOTERM_	GO:0045944~positive regulation of transcription from RNA polymerase II pror	33	11.37931	4.651353	3725, 10	259	981	16792	2.180959	0.091795	0.013660	0.080262
GOTERM_	GO:0045429~positive regulation of nitric oxide biosynthetic process	7	2.413793	4.697686	22914, 1	259	43	16792	10.55436	0.092665	0.012081	0.081061
GOTERM_	GO:0032496~response to lipopolysaccharide	12	4.137931	4.714702	3725, 11	259	164	16792	4.743949	0.092985	0.010785	0.081355
GOTERM_	GO:0008544~epidermis development	9	3.103448	4.831068	7357, 50	259	85	16792	6.864774	0.095167	0.009950	0.083362
GOTERM_	GO:0045766~positive regulation of angiogenesis	10	3.448275	7.073486	694, 801	259	115	16792	5.637737	0.136210	0.013223	0.122034
GOTERM_	GO:0007165~signal transduction	36	12.41379	9.864222	9982, 14	259	1161	16792	2.010355	0.184700	0.016872	0.170142
GOTERM_	GO:0071260~cellular response to mechanical stimulus	8	2.758620	1.072030	1647, 42	259	71	16792	7.305236	0.199022	0.016926	0.184895
GOTERM_	GO:0030335~positive regulation of cell migration	12	4.137931	1.326767	80310, 3	259	184	16792	4.228302	0.240168	0.019427	0.228783
GOTERM_	GO:0006366~transcription from RNA polymerase II promoter	21	7.241379	1.361093	3725, 67	259	513	16792	2.654022	0.245549	0.018609	0.234695
GOTERM_	GO:0008285~negative regulation of cell proliferation	18	6.206896	1.408314	3725, 67	259	396	16792	2.946998	0.252889	0.018056	0.242828
GOTERM_	GO:0045893~positive regulation of transcription, DNA-templated	21	7.241379	1.434190	3725, 68	259	515	16792	2.643715	0.256880	0.017313	0.247285
GOTERM_	GO:0007267~cell-cell signaling	14	4.827586	1.592497	9982, 19	259	254	16792	2821877	0.280841	0.018148	0.274545
GOTERM_	GO:0050728~negative regulation of inflammatory response	8	2.758620	2.105670	7538, 26	259	79	16792	6.565466	0.353330	0.022681	0.362864
GOTERM_	GO:0070301~cellular response to hydrogen peroxide	7	2.413793	2.336895	80310, 5	259	57	16792	7.962067	0.383559	0.023899	0.402635
GOTERM_	GO:0006954~inflammatory response	17	5.862068	2.644653	148022,	259	379	16792	2.908120	0.421615	0.025735	0.455546
GOTERM_	GO:0045892~negative regulation of transcription, DNA-templated	20	6.896551	2.694427	3725, 10	259	499	16792	2.598556	0.427545	0.025036	0.464101
GOTERM_	GO:0009612~response to mechanical stimulus	7	2.413793	2.828328	3725, 57	259	59	16792	7.692166	0.443198	0.025137	0.487112
GOTERM_	GO:0071356~cellular response to tumor necrosis factor	9	3.103448	2.959137	3037, 75	259	110	16792	5.304598	0.458077	0.025203	0.509587
GOTERM_	GO:0048661~positive regulation of smooth muscle cell proliferation	7	2.413793	3.102661	80310, 3	259	60	16792	7.563963	0.473945	0.025366	0.534240
GOTERM_	GO:0071222~cellular response to lipopolysaccharide	9	3.103448	3.553080	7538, 22	259	113	16792	5.163768	0.520790	0.027896	0.611573
GOTERM_	GO:0001822~kidney development	8	2.758620	000E-04	3037, 50	259	86	16792	6.031067	0.521851	0.026957	0.613410
GOTERM_	GO:0050927~positive regulation of positive chemotaxis	4	1.379310	5.399378	3791, 21	259	11	16792	23.57599	0.673058	0.039141	0.927974
GOTERM_	GO:0008283~cell proliferation	16	5.517241	5.589861	596, 291	259	366	16792	2.834272	0.685705	0.039125	0.960563
GOTERM_	GO:0010628~positive regulation of gene expression	13	4.482758	7.655190	639, 355	259	262	16792	3.216953	0.795099	0.051469	1.313266
GOTERM_	GO:0071347~cellular response to interleukin-1	7	2.413793	7.713060	3037, 80	259	71	16792	6.392082	0.797541	0.050218	1.323131
GOTERM_	GO:0009791~post-embryonic development	7	2.413793	8.937067	4633, 63	259	73	16792	6.216956	0.842888	0.056196	1.531580
GOTERM_	GO:0061029~eyelid development in camera-type eye	4	1.379310	9.148220	3725, 67	259	13	16792	19.94891	0.849613	0.055793	1.567497
GOTERM_	GO:0019221~cytokine-mediated signaling pathway	9	3.103448	9.484979	3448, 90	259	131	16792	4.454242	0.859749	0.056136	1.624755
GOTERM_	GO:0044344~cellular response to fibroblast growth factor stimulus	5	1.724137	0.001089	7538, 27	259	30	16792	10.80566	0.895278	0.062435	7529598
GOTERM_	GO:0002009~morphogenesis of an epithelium	4	1.379310	0.001151	54101, 7	259	14	16792	18.52399	0.907842	0.064083	1.968658
GOTERM_	GO:0002042~cell migration involved in sprouting angiogenesis	4	1.379310	0.001422	6722, 19	259	15	16792	17.28906	0.947505	0.076560	2.427665
GOTERM_	GO:0006959~humoral immune response	6	2.068965	0.001798	596, 262	259	57	16792	6.824629	0.975897	0.093385	3.058973
GOTERM_	GO:0072593~reactive oxygen species metabolic process	5	1.724137	0.001960	5076, 59	259	35	16792	9056812	0.982779	0.098904	3.330376
GOTERM_	GO:0071407~cellular response to organic cyclic compound	6	2.068965	0.002097	688, 229	259	59	16792	6.593285	0.987039	0.102951	3.559205
GOTERM_	GO:0007598~blood coagulation, extrinsic pathway	3	1.034482	0.002280	2158, 21	259	5	16792	1003861	0.991143	0.108885	3.864931
GOTERM_	GO:0045597~positive regulation of cell differentiation	5	1.724137	0.002413	3725, 67	259	37	16792	2080768	0.993281	0.112296	4.086234
GOTERM_	GO:0001525~angiogenesis	11	3.793103	0.002417	3725, 83	259	223	16792	3.198088	0.993332	0.109991	4.092331
GOTERM_	GO:0030890~positive regulation of B cell proliferation	5	1.724137	0.002935	596, 148	259	39	16792	8.312048	0.997722	0.129151	4.947544
GOTERM_	GO:0046330~positive regulation of JNK cascade	6	2.068965	0.003212	4616, 16	259	65	16792	5.984674	0.998718	0.137567	5.402479
GOTERM_	GO:0008584~male gonad development	7	2.413793	0.003273	221823,	259	94	16792	0465785	0.998872	0.137193	5.503445
GOTERM_	GO:0030336~negative regulation of cell migration	7	2.413793	0.003450	6722, 71	259	95	16792	4.777240	0.999219	0.141214	5.792285
GOTERM_	GO:0070527~platelet aggregation	5	1.724137	0.003528	10627, 1	259	41	16792	7.906582	0.999335	0.141380	5.919088
GOTERM_	GO:0071456~cellular response to hypoxia	7	2.413793	0.003634	3777, 59	259	96	16792	4.727477	0.999466	0.142566	7052286
GOTERM_	GO:0042730~fibrinolysis	4	1.379310	0.003885	5327, 53	259	21	16792	12.34932	0.999683	0.148844	9866019
GOTERM_	GO:0001836~release of cytochrome c from mitochondria	4	1.379310	0.005057	3725, 59	259	23	16792	11.27547	0.999972	0.186012	8.381017
GOTERM_	GO:0016477~cell migration	9	3.103448	0.005172	23237, 6	259	172	16792	3.392475	0.999978	0.186518	8.562779
GOTERM_	GO:0010595~positive regulation of endothelial cell migration	5	1.724137	0.005355	2625, 80	259	46	16792	7.047171	0.999985	0.189210	8.853676
GOTERM_	GO:0090102~cochlea development	4	1.379310	0.005715	3777, 50	259	24	16792	10.80566	0.999992	0.197259	9.420860
GOTERM_	GO:0051781~positive regulation of cell division	5	1.724137	0.005784	80310, 2	259	47	16792	6.897231	0.999993	0.196155	9.529657
GOTERM_	GO:0007623~circadian rhythm	6	2.068965	0.005931	3725, 10	259	75	16792	5.186718	0.999995	0.197419	9.760555
GOTERM_	GO:0007050~cell cycle arrest	8	2.758620	0.006163	1022, 58	259	141	16792	3.678523	0.999997	0.201102	10.12263
GOTERM_	GO:0030216~keratinocyte differentiation	6	2.068965	0.006271	1832, 30	259	76	16792	5.118471	0.999997	0.201121	10.29178
GOTERM_	GO:0000188~inactivation of MAPK activity	4	1.379310	0.006422	1843, 11	259	25	16792	10.37343	0.999998	0.202317	10.52558
GOTERM_	GO:0002053~positive regulation of mesenchymal cell proliferation	4	1.379310	0.007178	2294, 86	259	26	16792	9.974457	0.999999	0.220068	11.69404
GOTERM_	GO:0031018~endocrine pancreas development	4	1.379310	0.007178	301, 665	259	26	16792	9.974457	0.999999	0.220068	11.69404
GOTERM_	GO:0007189~adenylate cyclase-activating G-protein coupled receptor signalir	5	1.724137	0.007206	8698, 57	259	50	16792	6.483397	0.999999	0.217646	11.73781
GOTERM_	GO:0000165~MAPK cascade	11	3.793103	0.007462	2248, 75	259	262	16792	2.722037	0.999999	0.221254	12.12856
GOTERM_	GO:0043583~ear development	3	1.034482	0.007883	596, 262	259	9	16792	21.61132	0.999999	0.228997	12.77077
GOTERM_	GO:0010575~positive regulation of vascular endothelial growth factor produc	4	1.379310	0.007985	5743, 35	259	27	16792	9.605033	0.999999	0.228417	12.92477
GOTERM_	GO:0071333~cellular response to glucose stimulus	5	1.724137	0.008272	6722, 50	259	52	16792	6.234036	0.999999	0.232450	13.35935
GOTERM_	GO:0001701~in utero embryonic development	9	3.103448	0.008423	6722, 71	259	187	16792	3.120351	0.999999	0.233044	13.58710
GOTERM_	GO:0003281~ventricular septum development	4	1.379310	0.008843	639, 349	259	28	16792	9056812	0.999999	0.240001	14.21599
GOTERM_	GO:0060070~canonical Wnt signaling pathway	6	2.068965	0.009043	6659, 49	259	83	16792	4.686793	0.999999	0.241590	14.51359
GOTERM_	GO:0035556~intracellular signal transduction	14	4.827586	0.009691	7538, 29	259	403	16792	2.252296	0.999999	0.253364	15.47491
GOTERM_	GO:0048008~platelet-derived growth factor receptor signaling pathway	4	1.379310	0.009753	80310, 8	259	29	16792	8.942617	0.999999	0.251619	15.56566
GOTERM_	GO:0006928~movement of cell or subcellular component	6	2.068965	0.010454	7168, 80	259	86	16792	4.523300	0.999999	0.263911	16.59139
GOTERM_	GO:0001570~vasculogenesis	5	1.724137	0.010702	3037, 22	259	56	16792	5.788747	0.999999	0.266088	16.95207
GOTERM_	GO:0030513~positive regulation of BMP signaling pathway	4	1.379310	0.011732	3491, 32	259	31	16792	8.365674	0.999999	0.284406	18.43086
GOTERM_	GO:0048715~negative regulation of oligodendrocyte differentiation	3	1.034482	0.011803	11221, 3	259	11	16792	17.68199	0.999999	0.282610	18.53228
GOTERM_	GO:0001823~mesonephros development	3	1.034482	0.011803	5076, 26	259	11	16792	17.68199	0.999999	0.282610	18.53228
GOTERM_	GO:0035970~peptidyl-threonine dephosphorylation	3	1.034482	0.011803	1843, 18	259	11	16792	17.68199	0.999999	0.282610	18.53228
GOTERM_	GO:0007010~cytoskeleton organization	8	2.758620	0.012311	23237, 2	259	161	16792	3.221564	0.999999	0.289576	19.25187
GOTERM_	GO:0070830~bicellular tight junction assembly	4	1.379310	0.012801	6722, 57	259	32	16792	8.104247	0.999999	0.295959	19.94103
GOTERM_	GO:0032355~response to estradiol	6	2.068965	0.013129	1843, 30	259	91	16792	4.274767	0.999999	0.299045	20.39985
GOTERM_	GO:0006955~immune response	14	4.827586	0.013489	3493, 29	259	421	16792	2.155999	0.999999	0.302619	20.89882
GOTERM_	GO:0050679~positive regulation of epithelial cell proliferation	5	1.724137	0.013551	5076, 11	259	60	16792	5.402831	0.999999	0.300594	20.98539
GOTERM_	GO:0045732~positive regulation of protein catabolic process	5	1.724137	0.013551	10769, 5	259	60	16792	5.402831	0.999999	0.300594	20.98539
GOTERM_	GO:0009653~anatomical structure morphogenesis	6	2.068965	0.013715	51085, 4	259	92	16792	4.228302	0.999999	0.300469	21.21128
GOTERM_	GO:0071364~cellular response to epidermal growth factor stimulus	4	1.379310	0.013924	5076, 75	259	33	16792	7.858663	0.999999	0.301171	21.49898
GOTERM_	GO:0051607~defense response to virus	8	2.758620	0.013934	596, 148	259	165	16792	3.143465	0.999999	0.298286	21.51239
GOTERM_	GO:0002089~lens morphogenesis in camera-type eye	3	1.034482	0.014021	357, 421	259	12	16792	16.20849	0.999999	0.296839	21.63221
GOTERM_	GO:0050678~regulation of epithelial cell proliferation	3	1.034482	0.014021	4070, 32	259	12	16792	16.20849	0.999999	0.296839	21.63221
GOTERM_	GO:0071498~cellular response to fluid shear stress	3	1.034482	0.014021	3037, 76	259	12	16792	16.20849	0.999999	0.296839	21.63221
GOTERM_	GO:0001706~endoderm formation	3	1.034482	0.014021	1843, 18	259	12	16792	16.20849	0.999999	0.296839	21.63221
GOTERM_	GO:0097192~extrinsic apoptotic signaling pathway in absence of ligand	4	1.379310	0.015102	597, 596	259	34	16792	7.627526	0.999999	0.312708	23.10152
GOTERM_	GO:0009611~response to wounding	5	1.724137	0.015977	7538, 33	259	63	16792	5.145553	0.999999	0.324454	24.27261
GOTERM_	GO:0030501~positive regulation of bone mineralization	4	1.379310	0.016334	116039,	259	35	16792	7.409597	0.999999	0.327265	24.74529
GOTERM_	GO:0051092~positive regulation of NF-kappaB transcription factor activity	7	2.413793	0.016875	54101, 8	259	133	16792	3.412314	0.999999	0.332985	25.45687
GOTERM_	GO:0001666~response to hypoxia	8	2.758620	0.017141	6722, 49	259	172	16792	3.015533	0.999999	0.334167	25.80482
GOTERM_	GO:0042981~regulation of apoptotic process	9	3.103448	0.017360	1843, 88	259	213	16792	2.739463	0.999999	0.334571	26.08934
GOTERM_	GO:0043065~positive regulation of apoptotic process	11	3.793103	0.017962	1843, 46	259	300	16792	2.377245	1	0.340907	3772296
GOTERM_	GO:0031103~axon regeneration	3	1.034482	0.018946	3725, 83	259	14	16792	13.89299	1	0.352815	28.12230
GOTERM_	GO:0034605~cellular response to heat	4	1.379310	0.018962	1068, 26	259	37	16792	7.009078	1	0.349984	28.14233
GOTERM_	GO:0019933~cAMP-mediated signaling	4	1.379310	0.020359	108, 107	259	38	16792	6.824629	1	0.367352	29.88825
GOTERM_	GO:0042517~positive regulation of tyrosine phosphorylation of Stat3 protein	4	1.379310	0.020359	3280, 90	259	38	16792	6.824629	1	0.367352	29.88825
GOTERM_	GO:0008360~regulation of cell shape	7	2.413793	0.021156	10627, 7	259	140	16792	3.241698	1	0.375561	30.86685
GOTERM_	GO:0042633~hair cycle	3	1.034482	0.021642	5743, 38	259	15	16792	12.96679	1	0.379211	31.45693
GOTERM_	GO:0051091~positive regulation of sequence-specific DNA binding transcripti	6	2.068965	0.022978	6722, 84	259	105	16792	3.704798	1	0.394225	33.05391
GOTERM_	GO:0007507~heart development	8	2.758620	0.023185	4633, 67	259	183	16792	2.834272	1	0.393832	33.29821
GOTERM_	GO:0048468~cell development	4	1.379310	0.023318	84159, 8	259	40	16792	6.483397	1	0.392489	33.45582
GOTERM_	GO:0043280~positive regulation of cysteine-type endopeptidase activity invo	4	1.379310	0.023318	3491, 14	259	40	16792	6.483397	1	0.392489	33.45582
GOTERM_	GO:0043010~camera-type eye development	4	1.379310	0.023318	4081, 50	259	40	16792	6.483397	1	0.392489	33.45582
GOTERM_	GO:1900745~positive regulation of p38MAPK cascade	3	1.034482	0.024487	4616, 16	259	16	16792	12.15637	1	0.404517	34.81691
GOTERM_	GO:2001234~negative regulation of apoptotic signaling pathway	3	1.034482	0.024487	596, 492	259	16	16792	12.15637	1	0.404517	34.81691
GOTERM_	GO:0042127~regulation of cell proliferation	8	2.758620	0.024508	3725, 10	259	185	16792	2.803631	1	0.401695	34.84174
GOTERM_	GO:0001658~branching involved in ureteric bud morphogenesis	4	1.379310	0.026498	5076, 59	259	42	16792	6.174664	1	0.423292	37.09844
GOTERM_	GO:0048538~thymus development	4	1.379310	0.028171	6722, 59	259	43	16792	6.031067	1	0.440056	6697257
GOTERM_	GO:0030155~regulation of cell adhesion	4	1.379310	0.028171	6722, 39	259	43	16792	6.031067	1	0.440056	6697257
GOTERM_	GO:0006915~apoptotic process	16	5.517241	0.029349	4616, 52	259	567	16792	1.829530	1	0.450461	40.20387
GOTERM_	GO:0060532~bronchus cartilage development	2	0.689655	0.030493	6722, 66	259	2	16792	64.83397	1	0.460109	41.40871
GOTERM_	GO:0072179~nephric duct formation	2	0.689655	0.030493	5076, 26	259	2	16792	64.83397	1	0.460109	41.40871
GOTERM_	GO:0061145~lung smooth muscle development	2	0.689655	0.030493	6722, 66	259	2	16792	64.83397	1	0.460109	41.40871
GOTERM_	GO:0002690~positive regulation of leukocyte chemotaxis	3	1.034482	0.030601	2150, 35	259	18	16792	10.80566	1	0.458119	41.52095
GOTERM_	GO:0045216~cell-cell junction organization	3	1.034482	0.030601	55679, 1	259	18	16792	10.80566	1	0.458119	41.52095
GOTERM_	GO:0061436~establishment of skin barrier	3	1.034482	0.030601	2312, 38	259	18	16792	10.80566	1	0.458119	41.52095
GOTERM_	GO:0036120~cellular response to platelet-derived growth factor stimulus	3	1.034482	0.030601	80310, 3	259	18	16792	10.80566	1	0.458119	41.52095
GOTERM_	GO:0007275~multicellular organism development	15	5.172413	0.030893	27042, 5	259	521	16792	1.866621	1	0.458177	41.82451
GOTERM_	GO:0032755~positive regulation of interleukin-6 production	4	1.379310	0.031680	148022,	259	45	16792	5.763020	1	0.463565	42.63494
GOTERM_	GO:0071363~cellular response to growth factor stimulus	4	1.379310	0.031680	7050, 93	259	45	16792	5.763020	1	0.463565	42.63494
GOTERM_	GO:0030198~extracellular matrix organization	8	2.758620	0.032038	83700, 3	259	196	16792	2.646284	1	0.464265	42.99918
GOTERM_	GO:0051591~response to cAMP	4	1.379310	0.033517	1843, 37	259	46	16792	5.637737	1	0.476614	44.48447
GOTERM_	GO:0001894~tissue homeostasis	3	1.034482	0.033860	580, 149	259	19	16792	10.23694	1	0.477034	44.82402
GOTERM_	GO:0042060~wound healing	5	1.724137	0.034736	7032, 18	259	80	16792	4.052123	1	0.482785	45.68038
GOTERM_	GO:0071385~cellular response to glucocorticoid stimulus	3	1.034482	0.037249	7538, 30	259	20	16792	9.725096	1	0.504204	48.07060
GOTERM_	GO:0014911~positive regulation of smooth muscle cell migration	3	1.034482	0.037249	3037, 59	259	20	16792	9.725096	1	0.504204	48.07060
GOTERM_	GO:0051897~positive regulation of protein kinase B signaling	5	1.724137	0.040442	2625, 21	259	84	16792	3.859165	1	0.530572	50.96494
GOTERM_	GO:2000378~negative regulation of reactive oxygen species metabolic proces	3	1.034482	0.040761	5076, 59	259	21	16792	9.261996	1	0.530291	51.24540
GOTERM_	GO:0045987~positive regulation of smooth muscle contraction	3	1.034482	0.040761	6722, 57	259	21	16792	9.261996	1	0.530291	51.24540
GOTERM_	GO:0033198~response to ATP	3	1.034482	0.040761	1609, 35	259	21	16792	9.261996	1	0.530291	51.24540
GOTERM_	GO:0018149~peptide cross-linking	4	1.379310	0.041403	1832, 30	259	50	16792	5.186718	1	0.532862	51.80603
GOTERM_	GO:0051726~regulation of cell cycle	6	2.068965	0.042554	4616, 37	259	124	16792	3.137127	1	1834427	52.79552
GOTERM_	GO:0071277~cellular response to calcium ion	4	1.379310	0.043508	3725, 10	259	51	16792	5.085017	1	0.544795	53.60042
GOTERM_	GO:0010165~response to X-ray	3	1.034482	0.044392	301, 705	259	22	16792	4099684	1	0.549123	3169882
GOTERM_	GO:0046427~positive regulation of JAK-STAT cascade	3	1.034482	0.044392	3280, 15	259	22	16792	4099684	1	0.549123	3169882
GOTERM_	GO:0090103~cochlea morphogenesis	3	1.034482	0.044392	5076, 32	259	22	16792	4099684	1	0.549123	3169882
GOTERM_	GO:2000611~positive regulation of thyroid hormone generation	2	0.689655	0.045391	2625, 32	259	3	16792	43.22265	1	0.554279	55.15240
GOTERM_	GO:0070561~vitamin D receptor signaling pathway	2	0.689655	0.045391	5292, 15	259	3	16792	43.22265	1	0.554279	55.15240
GOTERM_	GO:0071409~cellular response to cycloheximide	2	0.689655	0.045391	9314, 46	259	3	16792	43.22265	1	0.554279	55.15240
GOTERM_	GO:0072189~ureter development	2	0.689655	0.045391	5076, 22	259	3	16792	43.22265	1	0.554279	55.15240
GOTERM_	GO:1990646~cellular response to prolactin	2	0.689655	0.045391	3569, 46	259	3	16792	43.22265	1	0.554279	55.15240
GOTERM_	GO:0003278~apoptotic process involved in heart morphogenesis	2	0.689655	0.045391	3491, 14	259	3	16792	43.22265	1	0.554279	55.15240
GOTERM_	GO:0001660~fever generation	2	0.689655	0.045391	3553, 35	259	3	16792	43.22265	1	0.554279	55.15240
GOTERM_	GO:0060675~ureteric bud morphogenesis	2	0.689655	0.045391	4070, 32	259	3	16792	43.22265	1	0.554279	55.15240
GOTERM_	GO:0010641~positive regulation of platelet-derived growth factor receptor sig	2	0.689655	0.045391	2152, 21	259	3	16792	43.22265	1	0.554279	55.15240
GOTERM_	GO:0060947~cardiac vascular smooth muscle cell differentiation	2	0.689655	0.045391	6722, 26	259	3	16792	43.22265	1	0.554279	55.15240
GOTERM_	GO:0034140~negative regulation of toll-like receptor 3 signaling pathway	2	0.689655	0.045391	7128, 21	259	3	16792	43.22265	1	0.554279	55.15240
GOTERM_	GO:0042472~inner ear morphogenesis	4	1.379310	0.045665	5076, 26	259	52	16792	4.987228	1	0.553485	55.37416
GOTERM_	GO:0045727~positive regulation of translation	4	1.379310	0.047874	6659, 38	259	53	16792	4.893130	1	0.567974	57.12440
GOTERM_	GO:0007565~female pregnancy	5	1.724137	0.048279	5676, 59	259	89	16792	3.642358	1	0.568121	57.43811
GOTERM_	GO:0048146~positive regulation of fibroblast proliferation	4	1.379310	0.050135	80310, 3	259	54	16792	4.802516	1	0.579216	58.84849
GOTERM_	GO:0060548~negative regulation of cell death	4	1.379310	0.052448	3491, 66	259	55	16792	4.715198	1	0.593163	60.54393
GOTERM_	GO:0043524~negative regulation of neuron apoptotic process	6	2.068965	0.053091	3725, 59	259	132	16792	2.946998	1	0.594810	61.00397
GOTERM_	GO:0070373~negative regulation of ERK1 and ERK2 cascade	4	1.379310	0.059687	1843, 26	259	58	16792	4.471308	1	0.636165	2655764
GOTERM_	GO:0045840~positive regulation of mitotic nuclear division	3	1.034482	0.060022	3553, 35	259	26	16792	8084348	1	0.635386	65.64874
GOTERM_	GO:0045986~negative regulation of smooth muscle contraction	2	0.689655	0.060061	5743, 15	259	4	16792	32.41698	1	0.632742	9698494
GOTERM_	GO:0035799~ureter maturation	2	0.689655	0.060061	5076, 26	259	4	16792	32.41698	1	0.632742	9698494
GOTERM_	GO:0035910~ascending aorta morphogenesis	2	0.689655	0.060061	6659, 32	259	4	16792	32.41698	1	0.632742	9698494
GOTERM_	GO:0010748~negative regulation of plasma membrane long-chain fatty acid t	2	0.689655	0.060061	8660, 70	259	4	16792	32.41698	1	0.632742	9698494
GOTERM_	GO:0044752~response to human chorionic gonadotropin	2	0.689655	0.060061	2729, 46	259	4	16792	32.41698	1	0.632742	9698494
GOTERM_	GO:0072289~metanephric nephron tubule formation	2	0.689655	0.060061	5076, 66	259	4	16792	32.41698	1	0.632742	9698494
GOTERM_	GO:0010735~positive regulation of transcription via serum response element	2	0.689655	0.060061	6722, 14	259	4	16792	32.41698	1	0.632742	9698494
GOTERM_	GO:0060534~trachea cartilage development	2	0.689655	0.060061	6722, 66	259	4	16792	32.41698	1	0.632742	9698494
GOTERM_	GO:0003170~heart valve development	2	0.689655	0.060061	639, 666	259	4	16792	32.41698	1	0.632742	9698494
GOTERM_	GO:0022028~tangential migration from the subventricular zone to the olfacto	2	0.689655	0.060061	6722, 65	259	4	16792	32.41698	1	0.632742	9698494
GOTERM_	GO:0070164~negative regulation of adiponectin secretion	2	0.689655	0.060061	3553, 80	259	4	16792	32.41698	1	0.632742	9698494
GOTERM_	GO:0071322~cellular response to carbohydrate stimulus	2	0.689655	0.060061	51085, 4	259	4	16792	32.41698	1	0.632742	9698494
GOTERM_	GO:0031394~positive regulation of prostaglandin biosynthetic process	2	0.689655	0.060061	301, 574	259	4	16792	32.41698	1	0.632742	9698494
GOTERM_	GO:0060349~bone morphogenesis	3	1.034482	0.064185	3037, 11	259	27	16792	7.203775	1	0.655100	68.18249
GOTERM_	GO:0001656~metanephros development	3	1.034482	0.064185	596, 339	259	27	16792	7.203775	1	0.655100	68.18249
GOTERM_	GO:0042476~odontogenesis	3	1.034482	0.064185	3399, 11	259	27	16792	7.203775	1	0.655100	68.18249
GOTERM_	GO:0042102~positive regulation of T cell proliferation	4	1.379310	0.064759	301, 328	259	60	16792	4.322265	1	0.655641	68.51729
GOTERM_	GO:0045669~positive regulation of osteoblast differentiation	4	1.379310	0.064759	3491, 34	259	60	16792	4.322265	1	0.655641	68.51729
GOTERM_	GO:0035335~peptidyl-tyrosine dephosphorylation	5	1.724137	0.066284	1843, 57	259	99	16792	3.274443	1	0.661665	4579163
GOTERM_	GO:0006469~negative regulation of protein kinase activity	5	1.724137	0.066284	4616, 16	259	99	16792	3.274443	1	0.661665	4579163
GOTERM_	GO:0006950~response to stress	4	1.379310	0.067367	4616, 11	259	61	16792	4.251408	1	0.665030	7214923
GOTERM_	GO:0043388~positive regulation of DNA binding	3	1.034482	0.068443	3280, 46	259	28	16792	6.946497	1	0.668275	70.59073
GOTERM_	GO:2000352~negative regulation of endothelial cell apoptotic process	3	1.034482	0.068443	2625, 71	259	28	16792	6.946497	1	0.668275	70.59073
GOTERM_	GO:0007616~long-term memory	3	1.034482	0.068443	6722, 10	259	28	16792	6.946497	1	0.668275	70.59073
GOTERM_	GO:0045087~innate immune response	12	4.137931	0.068693	3493, 63	259	430	16792	1.809320	1	0.666919	70.72703
GOTERM_	GO:0016337~single organismal cell-cell adhesion	5	1.724137	0.070251	1832, 84	259	101	16792	3.209602	1	0.672704	6876797
GOTERM_	GO:0042149~cellular response to glucose starvation	3	1.034482	0.072790	596, 801	259	29	16792	6.706963	1	0.683460	72.87212
GOTERM_	GO:0045214~sarcomere organization	3	1.034482	0.072790	6722, 71	259	29	16792	6.706963	1	0.683460	72.87212
GOTERM_	GO:0060412~ventricular septum morphogenesis	3	1.034482	0.072790	6659, 32	259	29	16792	6.706963	1	0.683460	72.87212
GOTERM_	GO:0002576~platelet degranulation	5	1.724137	0.074337	10447, 4	259	103	16792	3.147280	1	0.688743	73.64286
GOTERM_	GO:0001780~neutrophil homeostasis	2	0.689655	0.074506	83700, 3	259	5	16792	25.93359	1	0.686958	73.72574
GOTERM_	GO:0060591~chondroblast differentiation	2	0.689655	0.074506	3491, 82	259	5	16792	25.93359	1	0.686958	73.72574
GOTERM_	GO:0003211~cardiac ventricle formation	2	0.689655	0.074506	6659, 14	259	5	16792	25.93359	1	0.686958	73.72574
GOTERM_	GO:0051891~positive regulation of cardioblast differentiation	2	0.689655	0.074506	2627, 14	259	5	16792	25.93359	1	0.686958	73.72574
GOTERM_	GO:0072178~nephric duct morphogenesis	2	0.689655	0.074506	2625, 19	259	5	16792	25.93359	1	0.686958	73.72574
GOTERM_	GO:0031622~positive regulation of fever generation	2	0.689655	0.074506	5743, 35	259	5	16792	25.93359	1	0.686958	73.72574
GOTERM_	GO:2000379~positive regulation of reactive oxygen species metabolic proces	3	1.034482	0.077224	1647, 80	259	30	16792	6.483397	1	0.697859	75.02646
GOTERM_	GO:1900740~positive regulation of protein insertion into mitochondrial memb	3	1.034482	0.077224	596, 715	259	30	16792	6.483397	1	0.697859	75.02646
GOTERM_	GO:0033574~response to testosterone	3	1.034482	0.077224	1843, 70	259	30	16792	6.483397	1	0.697859	75.02646
GOTERM_	GO:0051017~actin filament bundle assembly	3	1.034482	0.077224	357, 443	259	30	16792	6.483397	1	0.697859	75.02646
GOTERM_	GO:0035019~somatic stem cell population maintenance	4	1.379310	0.078267	6659, 32	259	65	16792	8978319	1	0.700320	75.50938
GOTERM_	GO:0051384~response to glucocorticoid	4	1.379310	0.078267	1843, 59	259	65	16792	8978319	1	0.700320	75.50938
GOTERM_	GO:0043627~response to estrogen	4	1.379310	0.078267	2625, 76	259	65	16792	8978319	1	0.700320	75.50938
GOTERM_	GO:0014068~positive regulation of phosphatidylinositol 3-kinase signaling	4	1.379310	0.078267	80310, 3	259	65	16792	8978319	1	0.700320	75.50938
GOTERM_	GO:0043200~response to amino acid	3	1.034482	0.081741	1490, 35	259	31	16792	6.274255	1	0.714045	77.05464
GOTERM_	GO:0010332~response to gamma radiation	3	1.034482	0.081741	596, 262	259	31	16792	6.274255	1	0.714045	77.05464
GOTERM_	GO:0000187~activation of MAPK activity	5	1.724137	0.082861	3553, 18	259	107	16792	3.029625	1	0.716604	77.53336
GOTERM_	GO:0006936~muscle contraction	5	1.724137	0.082861	10627, 7	259	107	16792	3.029625	1	0.716604	77.53336
GOTERM_	GO:0006952~defense response	4	1.379310	0.083988	2625, 43	259	67	16792	8411802	1	0.719136	78.00509
GOTERM_	GO:0003007~heart morphogenesis	3	1.034482	0.086337	4633, 14	259	32	16792	6.078185	1	0.726914	78.95844
GOTERM_	GO:0045595~regulation of cell differentiation	3	1.034482	0.086337	9314, 58	259	32	16792	6.078185	1	0.726914	78.95844
GOTERM_	GO:0018108~peptidyl-tyrosine phosphorylation	6	2.068965	0.087324	2248, 37	259	153	16792	2.542508	1	0.728677	8724625
GOTERM_	GO:0051798~positive regulation of hair follicle development	2	0.689655	0.088730	10468, 3	259	6	16792	21.61132	1	0.732162	79.88983
GOTERM_	GO:2000347~positive regulation of hepatocyte proliferation	2	0.689655	0.088730	7128, 32	259	6	16792	21.61132	1	0.732162	79.88983
GOTERM_	GO:0002819~regulation of adaptive immune response	2	0.689655	0.088730	11221, 9	259	6	16792	21.61132	1	0.732162	79.88983
GOTERM_	GO:2000553~positive regulation of T-helper 2 cell cytokine production	2	0.689655	0.088730	2625, 35	259	6	16792	21.61132	1	0.732162	79.88983
GOTERM_	GO:2001054~negative regulation of mesenchymal cell apoptotic process	2	0.689655	0.088730	8200, 66	259	6	16792	21.61132	1	0.732162	79.88983
GOTERM_	GO:0003180~aortic valve morphogenesis	2	0.689655	0.088730	2625, 19	259	6	16792	21.61132	1	0.732162	79.88983
GOTERM_	GO:0060137~maternal process involved in parturition	2	0.689655	0.088730	1543, 19	259	6	16792	21.61132	1	0.732162	79.88983
GOTERM_	GO:0051150~regulation of smooth muscle cell differentiation	2	0.689655	0.088730	6722, 22	259	6	16792	21.61132	1	0.732162	79.88983
GOTERM_	GO:0006979~response to oxidative stress	5	1.724137	0.089557	1843, 69	259	110	16792	2.946998	1	2272577	80.20253
GOTERM_	GO:0001938~positive regulation of endothelial cell proliferation	4	1.379310	0.089883	3725, 37	259	69	16792	3.758491	1	0.732137	80.32465
GOTERM_	GO:0043154~negative regulation of cysteine-type endopeptidase activity invc	4	1.379310	0.089883	5076, 93	259	69	16792	3.758491	1	0.732137	80.32465
GOTERM_	GO:0010634~positive regulation of epithelial cell migration	3	1.034482	0.091008	3725, 17	259	33	16792	5.893997	1	0.734364	80.74045
GOTERM_	GO:0043491~protein kinase B signaling	3	1.034482	0.091008	5076, 35	259	33	16792	5.893997	1	0.734364	80.74045
GOTERM_	GO:0030855~epithelial cell differentiation	4	1.379310	0.092894	7380, 68	259	70	16792	3.704798	1	0.739576	81.41847
GOTERM_	GO:0071300~cellular response to retinoic acid	4	1.379310	0.092894	5076, 93	259	70	16792	3.704798	1	0.739576	81.41847
GOTERM_	GO:0032088~negative regulation of NF-kappaB transcription factor activity	4	1.379310	0.095946	80149, 7	259	71	16792	3.652618	1	0.749108	82.46864
GOTERM_	GO:0007411~axon guidance	6	2.068965	0.098809	9048, 26	259	159	16792	2.446565	1	0.757521	83.40291

Table S4 KEGG pathway of up-regulated genes												
Category	Term	Count	%	PValue	Genes	List Total	Pop Hits	Pop Total	Fold Enri	Bonferroi	Benjamin	FDR
KEGG_PA	hsa04064:NF-kappa B signaling pathway	9	3.103448	3.526536	597, 596	141	87	6910	5.069699	0.063829	0.063829	0.436269
KEGG_PA	hsa04668:TNF signaling pathway	9	3.103448	0.001318	3725, 88	141	106	6910	4.160979	0.218661	0.116066	1.622340
KEGG_PA	hsa04350:TGF-beta signaling pathway	8	2.758620	0.001488	7050, 33	141	84	6910	4.667342	0.243174	0.088691	1.829992
KEGG_PA	hsa04010:MAPK signaling pathway	14	4.827586	0.001903	3725, 67	141	255	6910	2.690585	0.299686	0.085206	2.333712
KEGG_PA	hsa04060:Cytokine-cytokine receptor interaction	13	4.482758	0.002343	3459, 34	141	230	6910	2.769966	1477103	0.084023	2.866997
KEGG_PA	hsa04610:Complement and coagulation cascades	7	2.413793	0.002612	2158, 53	141	69	6910	4.971733	0.386876	0.078296	3.190736
KEGG_PA	hsa05142:Chagas disease (American trypanosomiasis)	8	2.758620	0.004997	3725, 34	141	104	6910	3.769776	0.608186	0.125281	6.022061
KEGG_PA	hsa04530:Tight junction	9	3.103448	0.006474	4633, 83	141	137	6910	3.219444	0.703173	0.140862	7.735840
KEGG_PA	hsa05132:Salmonella infection	7	2.413793	0.006533	3725, 34	141	83	6910	4.133128	0.706471	0.127329	7.804136
KEGG_PA	hsa05202:Transcriptional misregulation in cancer	10	3.448275	0.006899	597, 435	141	168	6910	2.917088	0.726038	0.121444	8.224788
KEGG_PA	hsa05161:Hepatitis B	9	3.103448	0.009015	3725, 59	141	145	6910	3.041819	0.816138	0.142694	10.61920
KEGG_PA	hsa04913:Ovarian steroidogenesis	5	1.724137	0.016903	108, 574	141	49	6910	5.000723	0.958744	0.233304	19.04899
KEGG_PA	hsa05144:Malaria	5	1.724137	0.016903	22914, 3	141	49	6910	5.000723	0.958744	0.233304	19.04899
KEGG_PA	hsa05133:Pertussis	6	2.068965	0.017660	3725, 14	141	75	6910	3.920567	0.964277	0.226095	19.81806
KEGG_PA	hsa04024:cAMP signaling pathway	10	3.448275	0.018842	3725, 25	141	198	6910	2.475105	0.971478	0.224369	21.00573
KEGG_PA	hsa04510:Focal adhesion	10	3.448275	0.023687	80310, 3	141	206	6910	2.378985	0.988699	0.258336	25.70774
KEGG_PA	hsa05164:Influenza A	9	3.103448	0.024654	3725, 34	141	174	6910	2.534849	0.990611	0.253057	26.61510
KEGG_PA	hsa04923:Regulation of lipolysis in adipocytes	5	1.724137	0.026306	108, 574	141	56	6910	4.375633	0.993161	0.254158	28.14036
KEGG_PA	hsa04630:Jak-STAT signaling pathway	8	2.758620	0.027514	5292, 34	141	145	6910	2.703839	0.994578	0.251626	29.23809
KEGG_PA	hsa04932:Non-alcoholic fatty liver disease (NAFLD)	8	2.758620	0.033303	3725, 51	141	151	6910	3568644	0.998224	0.283490	34.28643
KEGG_PA	hsa05321:Inflammatory bowel disease (IBD)	5	1.724137	0.040255	3725, 34	141	64	6910	3.828679	0.999539	0.318989	39.91021
KEGG_PA	hsa04115:p53 signaling pathway	5	1.724137	0.046388	4616, 16	141	67	6910	3.657245	0.999861	0.344900	44.50039
KEGG_PA	hsa04380:Osteoclast differentiation	7	2.413793	0.049555	3725, 34	141	131	6910	2.618699	0.999925	0.350802	46.74249
KEGG_PA	hsa05219:Bladder cancer	4	1.379310	0.049603	7380, 70	141	41	6910	4.781179	0.999926	0.338763	46.77562
KEGG_PA	hsa04976:Bile secretion	5	1.724137	0.050750	28234, 4	141	69	6910	3.551238	0.999941	0.333569	47.56667
KEGG_PA	hsa04022:cGMP-PKG signaling pathway	8	2.758620	0.051185	6722, 48	141	166	6910	2.361787	0.999945	0.324979	47.86343
KEGG_PA	hsa04068:FoxO signaling pathway	7	2.413793	0.054234	4616, 10	141	134	6910	2.560071	0.999970	0.330379	49.90250
KEGG_PA	hsa05140:Leishmaniasis	5	1.724137	0.055330	3725, 34	141	71	6910	3.451203	0.999976	0.325798	50.61789
KEGG_PA	hsa05206:MicroRNAs in cancer	11	3.793103	0.063146	5292, 15	141	285	6910	1.891501	0.999994	0.353143	55.45027
KEGG_PA	hsa04620:Toll-like receptor signaling pathway	6	2.068965	0.063510	3725, 14	141	106	6910	2.773986	0.999995	0.344997	55.66439
KEGG_PA	hsa05146:Amoebiasis	6	2.068965	0.063510	107, 355	141	106	6910	2.773986	0.999995	0.344997	55.66439
KEGG_PA	hsa04261:Adrenergic signaling in cardiomyocytes	7	2.413793	0.075558	4633, 71	141	146	6910	2.349655	0.999999	0.387206	62.23914
KEGG_PA	hsa04925:Aldosterone synthesis and secretion	5	1.724137	0.081431	3777, 57	141	81	6910	3.025129	0.999999	0.400928	65.10752
KEGG_PA	hsa04151:PI3K-Akt signaling pathway	12	4.137931	0.090176	80310, 2	141	345	6910	1.704594	0.999999	0.424355	69.00918
KEGG_PA	hsa04670:Leukocyte transendothelial migration	6	2.068965	0.090908	4633, 83	141	118	6910	2.491886	0.999999	0.417304	69.31682
KEGG_PA	hsa05134:Legionellosis	4	1.379310	0.095789	2919, 35	141	54	6910	3.630154	0.999999	0.425246	71.29759
KEGG_PA	hsa04970:Salivary secretion	5	1.724137	0.096403	483, 108	141	86	6910	2.849249	0.999999	0.418193	1790749
KEGG_PA	hsa04621:NOD-like receptor signaling pathway	4	1.379310	0.099885	7128, 35	141	55	6910	3.564152	0.999999	0.421100	72.86806
